# The Role of γ-Aminobutyric Acid (GABA) in the Occurrence of Adventitious Roots and Somatic Embryos in Woody Plants

**DOI:** 10.3390/plants11243512

**Published:** 2022-12-14

**Authors:** Lu Pei, Yue Zhao, Xinru Shi, Rongrong Chen, Jiawei Yan, Xu Li, Zeping Jiang, Junhui Wang, Shengqing Shi

**Affiliations:** 1 State Key Laboratory of Tree Genetics and Breeding, Research Institute of Forestry, The Chinese Academy of Forestry, Beijing 100091, China; 2 College of Biological and Environmental Sciences, Zhejiang Wanli University, Ningbo 315100, China; 3 Key Laboratory of Forest Ecology and Environment of National Forestry and Grassland Administration, Research Institute of Forest Ecology, Environment and Protection, The Chinese Academy of Forestry, Beijing 100091, China

**Keywords:** GABA, adventitious rooting, somatic embryo, phytohormone, carbon and nitrogen metabolism

## Abstract

The occurrence of adventitious roots and somatic embryos is a crucial step in micropropagation that frequently limits the application of this technique in woody plants. Recent studies demonstrated that they can be negatively or positively regulated with γ-aminobutyric acid (GABA), which is a four-carbon non-proteinous amino acid that not only acts as a main inhibitory neurotransmitter in mammals. It has been reported that GABA affects plant growth and their response to stress although its mode of action is still unclear. This review dealt with the effects of GABA on adventitious root formation and growth as well as on somatic embryogenesis. Furthermore, we focused on discussing the interaction of GABA with phytohormones, such as auxin, ethylene, abscisic acid, and gibberellin, as well as with the carbon and nitrogen metabolism during adventitious root development. We suggested that research on GABA will contribute to the application of micropropagation in the recalcitrant fruit and forest species.

## 1. Introduction

Adventitious roots (ARs) and somatic embryos (SEs) are not only key processes in plant organ differentiation. They are also important for vegetative propagation and plant regeneration, allowing plant growth and adaptation to various environmental conditions. ARs arise on organs other than the primary root, such as plant leaves, stems, or embryonic axes. ARs can be formed in two ways: (i) direct organogenesis through established cell types (such as cambium) [1] and (ii) indirect organogenesis from the callus formed after mechanical damage, such as that which happens in ex vitro cutting and in tissue cultures [1]. ARs on the young stems of dicotyledons and gymnosperms are usually produced by pericycle cells, while those on older stems are produced by other tissues that are close to the vascular tissues, such as phloem parenchyma cells, xylem parenchyma cells, or interfascicular cambium cells [2,3]. Some plants do not easily form ARs. This process is regulated by internal and external factors, such as hormones [2,4,5,6] and the carbon/nitrogen metabolism [5,7,8,9,10]. Treatment with auxin can promote AR formation [11], while gibberellin (GA) generally has a contrary role [12]. In addition, other compounds have a potential role in AR formation, such as γ-aminobutyric acid (GABA) [5,13], which has been localized in vascular tissues, including differentiating xylem and ray parenchymas [14].

The occurrence of SEs is the process of the differentiation of cells with a bipolar structure resembling a zygotic embryo into a plant [15], which is derived from somatic cells and is dependent on the totipotency of plant cells. Somatic cells transform into embryonic cells through de-differentiation and re-differentiation and then regenerate somatic embryos to produce intact plants [16] mainly through five steps: somatic embryo induction, proliferation, maturation, plantlet conversion, and subsequent plant acclimatization [17]. According to the occurrence modes, there are two pathways: (i) direct embryogenesis in which the cultivation of explants from microspores, ovules, embryos, and seedlings on media containing the appropriate balance of plant growth regulators can induce the formation of embryos directly (direct SE) and (ii) indirect embryogenesis in which the explants produce a callus that will eventually produce somatic embryos [15]. This process is influenced by many factors, such as auxins and cytokinins as reported for European chestnuts [18], partial desiccation as reported for spruces [19], and exogenous GABA as reported for *Acca sellowiana* [20] and *Liriodendron* hybrids [21].

GABA is a four-carbon non-proteinous amino acid that is widely present in plants, and it plays an important role in the neuronal development of animals, plant defense, and microbial nutrient storage [22,23]. The existence of GABA in plants was first discovered in potato tubers in 1949 [24], and then it was detected in different parts of a variety of plants [25,26]. It is mainly synthesized and metabolized by a GABA shunt, which bypasses two steps in the tricarboxylic acid cycle (TCA cycle) in both animals and plants [27]. Specifically, α-ketoglutarate is converted to glutamate, which is then decarboxylated to form GABA by glutamate decarboxylase (GAD). The GABA is subsequently converted to succinic semialdehyde and succinate by GABA transaminase (GABA-T) and succinic semialdehyde dehydrogenase (SSADH), respectively [22,27]. Therefore, GABA is closely related to the TCA cycle and the carbon and nitrogen metabolism.

When plants encounter abiotic and biotic stresses, they rapidly accumulate GABA [28,29,30]. The increase in GABA provides sufficient carbon/nitrogen sources for the TCA cycle and amino acid synthesis [31,32,33]. However, GABA acts not only as a metabolite but also as a signaling molecule [27,34], which was proven by the recent identification of a GABA receptor, aluminum-activated malate transporter (ALMT) [35,36]. The recent genetic and physiological studies imply that GABA is required not only for responses to adverse conditions, such as abiotic stresses [31,37,38,39,40], biotic stresses [41,42], and hormone homeostasis [43,44], but also for developmental processes, such as pollen tube growth [45], stem growth [46], and primary root growth [25,47]. We found that the inhibition or delay of AR development in poplars is closely associated with GABA due to its interaction with hormone-related pathways as well as the carbon/nitrogen metabolism [5,9], proving that GABA may participate in the regulation of the AR occurrence in woody plants. We found that GABA can be negatively or positively related to ARs [5,9]. It has also been reported to have a positive association with SEs [20,21]. Treatment with a high GABA concentration (10 mM) negatively regulated AR formation and growth in poplars [5], and the same interaction has been reported in *Malus xiaojinensis* and tobacco plants [13]. A lower GABA concentration (10 µM), however, increased the length of poplar ARs [5], which was in agreement with the subsequent report that GABA (~60 µM) increased the root length of plantlets germinated from the somatic embryos of hybrid *Liriodendron* [21], whereas the effect of exogenous GABA (2 mM) on the primary root growth in *Brassica napus* seedlings was not significant [48]. These studies suggested that the effects of GABA on rooting could mainly be associated with differences in the dosage of GABA concentrations.

Since the formation of ARs and SEs are key steps in micropropagation and often limit the application of this technique to woody plants, it can be useful to investigate the relationship between GABA and both processes. However, the specific functions and roles of GABA in plants are still unanswered. In this review we discussed the interaction of GABA with phytohormones as well as with the carbon and nitrogen metabolism and its effects on the AR and SE occurrences with a special focus on woody plants.

## 2. GABA and Phytohormones during AR Development

Phytohormones are trace organic substances that regulate plant growth and development. GABA is closely related to a variety of hormones, and they interact with each other, suggesting that GABA may act as a signaling substance that affects phytohormones [5]. For example, exogenous GABA can induce the production of ethylene in *Caragana intermedia* [49] as well as abscisic acid (ABA) [43] and auxin IAA (3-indoleacetic acid) [5] in poplars (*Populus alba × P. glandulosa* cv. ‘84K’) (Table 1).

### 2.1. Auxin

The formation of ARs depends on a variety of factors among which phytohormones, especially auxin, play a key role. The application of the IAA and auxin precursor (IBA; 3-indolebutyric acid) induces AR formation during the micropropagation of woody plants [55]. Recent studies demonstrate that GABA is closely related to AR formation and growth due to its interaction with auxin [5,13]. Xie et al. [5] studied the influence of GABA on the rooting of poplars grown on 1/2 MS medium without auxin addition. As explants, they used the leafy stem segments from 1 month in vitro plantlets and propagated them with or without GABA for 12 d. They observed that 10 mM GABA delayed the initiation of AR primordia and inhibited the increase in root length at 1–3 d and 12 d, respectively, accompanied by a large endogenous IAA accumulation at 1–3 d compared to the control [5]. Li et al. [13] used a similar system with the rooting-recalcitrant plant *M. xiaojinensis*, grown on MS medium containing 0.5 mg/L IBA, to study the effects of GABA on rooting. They found that exogenous GABA (1–16 mM) inhibited AR formation by delaying root emergence and reducing root growth. This treatment also restrained the expression of auxin export carrier *PIN* family members at 1–4 d after root induction compared with the effect of IBA alone [13]. In poplars (as in most plants), an increase in IAA levels is required for AR induction although maintaining high levels for some days inhibits AR initiation and expression [2]. It seems that GABA can increase IAA levels in poplars over the physiologically required levels and perturb the polar auxin transport in *M. xiaojinensis* by inhibiting the auxin-induced upregulation of *PIN* family members, which have been positively associated with the process of AR formation in *M. domestica* [56]. Interestingly, despite the differences in rooting between poplars and *M. xiaojinensis*, it seems that there is a general trend in which treatments with high GABA concentrations inhibit the AR occurrence. This might occur by altering auxin homeostasis and distribution during the phases of initiation and expression in the early days of the rooting process. The exact action modes still need a systematic investigation.

### 2.2. Abscisic Acid and Ethylene

ABA and ethylene are two stress-related hormones, and they have been related to the AR occurrence [57,58,59,60]. The addition of GABA stimulated ethylene biosynthesis in sunflower tissues excised from 6 to 8 d old seedlings in a growth chamber [50] and increased ABA and ethylene in 6-week-old poplar in vitro plants [43] as well as ethylene in 3-week-old seedlings of *C. intermedia* cultured under NaCl stress [49]. During the inhibition of poplar AR formation, shoots treated with exogenous GABA showed an increased ABA concentration and a decreased ethylene level in the first day of treatment. The addition of γ-vinyl-γ-aminobutyric acid (VGB), a compound that blocks GABA degradation, caused a similar effect [5]. Yue et al. [9], using the same experimental system, treated poplar shoots with succinyl phosphate, which increased the activity of the GABA shunt by blocking α-ketoglutarate dehydrogenase activity. They found a significant decrease in the number and length of ARs together with a decrease in the ethylene concentration [9]. In addition, the application of GABA decreased ethylene release and inhibited the respiration rate during apple storage [51]. These studies indicated that both ethylene and ABA are related to GABA responses and the AR occurrence even if it is not clear which compound is the main driver of these responses.

### 2.3. Gibberellin

Gibberellin (GA) is a class of phytohormones belonging to diterpenoids which can induce flowering and break dormancy [61] and also inhibit AR growth in poplars [12,62]. However, the inhibitory effect of GA treatment in ARs is not mediated by the perturbation of the auxin signaling pathway but appears to act by perturbing polar auxin transport [12]. Interestingly, when the effects of GABA on the rooting of woody plants were investigated, it was found that GABA enhanced GA accumulation in poplars [5,9] and inhibited polar auxin transport in *M. xiaojinensis* [13], indicating a possible interaction of GABA with GA and auxin as well as other phytohormones during AR formation.

## 3. GABA and Carbon/Nitrogen Metabolism during AR Development

Carbon and nitrogen metabolism are closely related to AR formation [7,63,64]. GABA has been considered as an important component of the balance between carbon and nitrogen pools in plant cells [65,66]. Therefore, recent studies have pointed out that GABA can influence AR formation and growth by affecting the carbon and nitrogen metabolism [5,9] (Table 1).

### 3.1. Carbon Metabolism

The carbon metabolism is the most important basal metabolism in plants, providing the essential carbon framework and energy for the synthesis of amino acids, proteins, and nucleic acids in the nitrogen metabolism. One of the functions of GABA accumulation under adverse conditions is to provide an additional carbon source for the TCA cycle, ensuring the uninterrupted TCA cycle and alleviating the inhibitory effect of stress [31,32]. GABA contents are related to the concentration of organic acids, such as malate, citrate, and succinate in the TCA cycle [31,67]. During the inhibition of poplar AR growth, the levels of these three organic acids in the roots were significantly increased by treatment with 10 mM GABA [5]. In addition, after inhibiting GABA degradation with succinyl phosphate, malate was sharply increased in the roots and stems. Meanwhile succinate underwent significant changes in the stems, and citrate did not show changes [9]. Exogenous GABA and GABA-T inhibitor (AOA or Vir) treatments increased the effusion of malate and citrate as well as the relative root elongation rate in hybrid *Liriodendron* cultured under aluminum stress [52]. Under the same stress, the malate content in wheat was closely related to GABA, which exerted its physiological effects via the receptor ALMT, including the regulation of the pollen tube and root growth [35,36]. Thus, the current studies may suggest that the interaction of GABA and malate jointly affect AR formation and growth through the malate transporter ALMT.

Sugars can affect plant hormone synthesis and signaling [68]. The initial sugar content in the stems was positively correlated with the survival rates and AR formation [69,70,71]. During the inhibition of poplar AR formation and growth, exogenous GABA and VGB treatments produced a significant temporary increase of the sugar contents in the stems at 3 d followed by a decrease in that of the roots at 12 d [5]. Yue et al. [9] also found that the sugar contents decreased dramatically in the stems and roots of succinyl-phosphate-treated poplar shoots. Despite the discrepancies in the sugar responses found between the early and late phases in these two experiments [5,9], there was a general trend in which a high GABA concentration inhibited AR formation, and it was associated with changes in the homeostasis of sugar levels at the different phases of rooting.

### 3.2. Nitrogen Metabolism

The formation of ARs is dependent on the accumulation of the amino acids required for protein synthesis [52]. Xie et al. [5] found that the inhibition of AR formation and growth due to GABA treatment was associated with increased levels of amino acids in poplars, which are important resources for root development. Similarly, the application of succinyl phosphate resulted in significant increases in glutamate and GABA, key components of the GABA shunt, as well as most of the other detected amino acids in the inhibited poplar ARs at 12 d [9]. Du et al., however, studied the effects of applying exogenous GABA (10 mM) to chestnut seeds for 5 d and found a decrease in the content of most of the detected amino acids at 2 d, which was when the germination and the growth of the early primary roots were inhibited [53]. The discrepancies in the amino acid changes in these experiments may be related to the differences in the plant materials or tissues. A recent study on tea plants further demonstrated the involvement of GABA in the nitrogen metabolism. An exogenous GABA application increased the endogenous GABA levels and improved cold tolerance at the same time that the levels of low-temperature stress-responsive substances, such as glutamate, polyamines and anthocyanins, were altered [54]. Thus, the perturbation of the nitrogen metabolism by the addition of GABA might contribute to the inhibition of AR formation and growth as well as to other stress responses in plants, indicating that the GABA-inhibited AR occurrence in woody plants might be related to the direct effect of GABA interacting with phytohormones and also to the disruption of the carbon and nitrogen balance.

## 4. GABA and Somatic Embryogenesis

**Figure 1:**
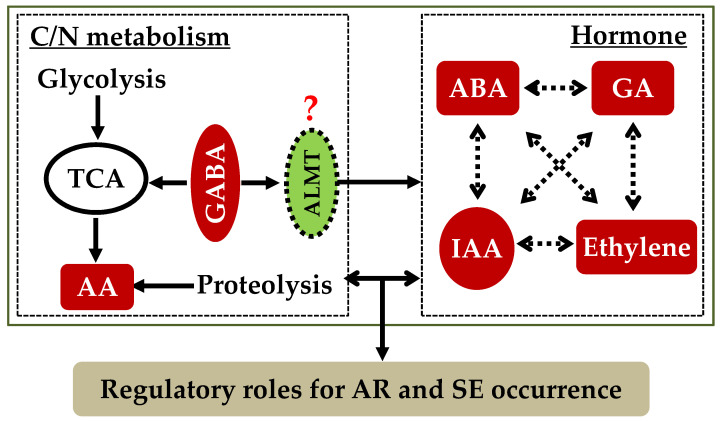
Hypothetical model for the roles of GABA in the occurrence of adventitious roots (ARs) and somatic embryos (SEs) due to its alteration of carbon and nitrogen metabolism and hormone homeostasis as well as their downstream signaling cascades, which was edited from that of Xie et al. (2020).

An earlier study on the somatic embryogenesis of *A. sellowiana* showed that endogenous GABA reached its highest concentration at 9 d and then decreased with fluctuations for 30 days [20]. The treatment of *A. sellowiana* zygotic embryos with 10 µM GABA significantly enhanced the induction rate of SEs [20]. Chen et al. [21] used a similar system with the embryogenic callus of hybrid *Liriodendron* to study the effects of GABA on the SE occurrence and found that GABA (~60 µM) showed a positive effect on the induction and maturation of SEs. They also observed an increase in the root length of plantlets germinated from the somatic embryos of hybrid *Liriodendron* [21] which was similar to the effect of a lower GABA concentration (10 µM) on the growth of ARs [5]. Moreover, exogenous GABA improved the quality of the somatic embryos and decreased the proportion of abnormal ones [20,21]. These findings suggested that GABA may play a key role in the SE occurrence in woody plants (Table 1), which may share speculated pathways similar to those of the AR occurrence due to interactions with phytohormones and the carbon and nitrogen metabolism (Figure 1) although the mode of action can be different.

## 5. Future Outlook

The current studies have proven that high GABA concentrations participate in the negative regulation of AR formation and growth, while lower GABA levels promote AR growth as well as SE induction and maturation in woody plants. These functions may be performed through interactions with phytohormones and alterations in the balance of the carbon/nitrogen metabolism (Figure 1) although its mode of action seems to be different in the occurrence of ARs compared to that of SEs. However, the accurate mechanism of regulation of GABA is unknown. Therefore, the overexpression or knockout of the key genes associated with the GABA shunt, such as *GAD*s and *GABA-T*s as well as the receptor *ALMT*, could be used to investigate the effects of GABA on the occurrence of ARs and SEs and could be combined with transcriptome and metabolome studies in the future. This could be beneficial in deciphering whether GABA is one of the key limiting factors for the propagation of woody plants, which would be a better supplement for understanding the mechanism of the AR and SE occurrences and for contributing to the application of asexual reproduction in recalcitrant fruit and forest species.

## Figures and Tables

**Table 1:** Effects of γ-aminobutyric acid (GABA) on the occurrence of adventitious roots (ARs) and somatic embryos (SEs).

Factors		Plant Species	Response	References
Phytohormones	Auxin	*Malus xiaojinensis*	High GABA concentrations inhibited AR formation and inhibited the expression of *PIN*s (auxin export carriers), therefore perturbing polar auxin transport.	[13]
		*Populus alba* × *Populus glandulosa* cv. ‘84K’	GABA (10 mM) delayed the initiation of AR primordia by 1–3 d and was accompanied by a large endogenous IAA accumulation at 1–3 d compared to the control.	[5]
	Ethylene	*Helianthus annuus*	GABA could stimulate ethylene biosynthesis.	[50]
		*Caragana intermedia*	GABA could increase ethylene biosynthesis under NaCl stress.	[49]
		*Populus tomentosa*	GABA had a positive effect on the increase in ethylene under NaCl stress.	[43]
		*Malus domestica* cv. Golden Delicious	GABA caused a decrease in ethylene during apple storage.	[51]
		*Populus alba* × *Populus glandulosa* cv. ‘84K’	GABA decreased the ethylene level at 1 d as well as the addition of γ-vinyl-γ-aminobutyric acid (VGB) during AR formation.	[5]
		*Populus alba* × *Populus glandulosa* cv. ‘84K’	The activity of the GABA shunt was increased after treatment with succinyl phosphate, and the growth of ARs (including root length and number) was significantly inhibited at 12 d. Ethylene was also reduced in the stems.	[9]
	Abscisic Acid (ABA)	*Populus tomentosa*	GABA had a positive effect on the increase in ABA under salt stress.	[43]
		*Populus alba* × *Populus glandulosa* cv. ‘84K’	GABA increased the ABA level in the early phase of AR formation.	[5]
	Gibberellin (GA)	*Populus alba* × *Populus glandulosa* cv. ‘84K’	GABA negatively regulated AR formation, which was accompanied by a high GA accumulation.	[5]
Carbon/nitrogen metabolism		*Populus alba* × *Populus glandulosa* cv. ‘84K’	GABA (10 mM) inhibited poplar AR formation and growth at 12 d, which was accompanied by a significant reduction in the sugar content and a significant increase in the amino acid and organic acid levels in roots.	[5]
		*Populus alba* × *Populus glandulosa* cv. ‘84K’	Succinyl phosphate inhibited AR growth at 12 d; meanwhile, the sugars in the stems and roots significantly decreased, and the amino acids and malates significantly increased.	[9]
		*Liriodendron chinense* *× tulipifera*	GABA and the GABA transaminase (GABA-T) inhibitor (AOA or Vir) increased the effusion of malate and citrate as well as the relative root elongation rate.	[52]
		*Castanea mollissima*	GABA (10 mM) inhibited the germination of chestnut seeds and the growth of early primary roots with a change in the carbon and nitrogen balance.	[53]
		*Camellia sinensis*	GABA induced interactions between photosynthesis, amino acid biosynthesis, and the carbon and nitrogen metabolism and improved cold tolerance.	[54]
Somatic embryogenesis occurrence		*Acca sellowiana*	GABA (10 µM) promoted the induction of somatic embryos and decreased the rate of abnormal ones.	[20]
		*Liriodendron* hybrid	GABA (~60 µM) enhanced the induction and maturation of somatic embryos and increased the root length of plantlets germinated from somatic embryos.	[21]

## Data Availability

No applicable.
